# Activity behaviours before and during pregnancy are associated with women’s device-measured physical activity and sedentary time in later parenthood: a longitudinal cohort analysis

**DOI:** 10.1123/jpah.2022-0630

**Published:** 2023-08-12

**Authors:** Kathryn R. Hesketh, Janis Baird, Sarah R. Crozier, Keith M. Godfrey, Nicholas C. Harvey, Cyrus Cooper, Esther M.F. van Sluijs

**Affiliations:** 1 MRC Epidemiology Unit, Institute of Metabolic Science, University of Cambridge School of Clinical Medicine, Cambridge, United Kingdom; 2 MRC Lifecourse Epidemiology Centre, University of Southampton, Southampton General Hospital, Southampton, United Kingdom; 3 NIHR Southampton Biomedical Research Centre, University of Southampton and University Hospital Southampton NHS Foundation Trust, Southampton, United Kingdom; 4 NIHR Applied Research Collaboration Wessex, Southampton Science Park, Southampton, United Kingdom

**Keywords:** Physical activity, sedentary, pre-conception, pregnancy, parenthood

## Abstract

**Purpose:**

To explore how activity behaviours before/during pregnancy relate to those in later parenthood, we assessed associations between sitting and moderate-/strenuous exercise before/during pregnancy, and sedentary time (SED) and moderate-to-vigorous physical activity (MVPA) 4-7 years postpartum (‘later parenthood’).

**Methods:**

Longitudinal data were from the Southampton Women’s Survey, UK. Women reported time spent sitting (hours/day), in moderate-strenuous exercise (hours/week), and in strenuous exercise (hours/week) at 3 time points before/during pregnancy (i.e. pre-conception, at ~12weeks and ~34weeks gestation). From this, we derived three behaviour trajectories for each woman. In later parenthood, women wore an accelerometer for ≤7 days (mean: 5.4(SD:1.8)days) which we used to derive two outcomes: minutes/day SED and in MVPA. Multi-level linear regression was used to explore associations between trajectories before/during pregnancy and device-measured SED/MVPA in later parenthood.

**Results:**

780 women provided valid data before/during pregnancy and in later parenthood. Consistent high sitters (vs. low) were more sedentary 4-7 years postpartum (β=39.5 minutes/day [95%CI: 23.26,55.82]), as were women in groups who sat more in later pregnancy. Consistently high moderate/-strenuous exercisers (vs. low) were 22% (95%CI: 2-47%) more active in later parenthood; those engaging in strenuous activity preconception tended to have higher MVPA as parents.

**Conclusions:**

Trajectories of sitting and exercise before/during pregnancy are associated with sedentary time and MVPA respectively in later parenthood. Interventions to reduce sitting in pregnancy, and to encourage higher intensity activity preconception, may benefit maternal and child health.

## Introduction

Physical activity confers benefits to physical and mental health,^1,2^ including during the pre-conception period, pregnancy and postpartum.^3,4^ In the UK, all women, including those who are pregnant or ≥6 weeks postpartum, are encouraged to engage in at least 150 minutes of moderate physical activity every week, and limit periods of extended sitting.^2,5^ Regular physical activity for women with uncomplicated pregnancies is associated with better weight management and physical fitness, decreased risk of gestational diabetes, and improved mental wellbeing,^3,6^ with lower risk of having a large-for-gestational-age infant.^6^ Conversely, increased sedentary time during pregnancy is linked to higher LDL cholesterol and C reactive protein in mothers, and larger newborn abdominal circumference and macrosomia in infants.^7^ Though women should be aware of medical contraindications,^3^ those who were physically active prior to pregnancy can continue to be so, and those who lead more sedentary lives also benefit from gradual increases in physical activity.^3,8^ Postpartum, returning to physical activity is associated with reduced risk of depression, improved emotional well-being and physical conditioning, and reduced postpartum weight gain, with a faster return to pre-pregnancy weight.^9^

In recent years, the preconception period (generally defined as a period prior to a (planned) pregnancy)^10^ has also gained prominence as a key period for promoting behaviour change,^11^ with evidence showing that preconception health and behaviours have a large impact on subsequent parental and infant health.^11^ However, whilst monitoring behaviours such as smoking and nutrition have been integrated into national preconception surveillance metrics,^12^ physical activity data remain largely absent, in part because they are not routinely collected during antenatal visits. Population-level data suggest that 40% of women aged 16-45 years (i.e. broadly of childbearing age) often fail to meet recommended levels of physical activity,^13,14^ and few studies have assessed activity behaviours (e.g. sedentary time, physical activity) from pre-conception through postpartum. Several studies using self-reported activity levels suggest that women engage in low levels of physical activity before and during pregnancy and do not meet physical activity guidelines;^15–18^ estimates of sitting or sedentary time tend to be of 4 hours or more hours/day during pregnancy.^19–21^ Limited evidence using device-based measures (e.g., accelerometers) suggests that activity levels are even lower than those self-reported.^22,23^ Although activity levels rebound to some extent post birth, they tend to remain low during the postpartum period,^24,25^ and may take years to return to pre-pregnancy levels.^19^ This is borne out by systematic review evidence which suggests that the transition to parenthood for women is associated with a decline in activity levels relative to their childless counterparts.^26,27^

Although limited evidence is available to determine how activity behaviours track as women transition into parenthood, less is known about the extent to which a woman’s activity behaviours before parenthood (pre-conception and during pregnancy – hereafter ‘before/during pregnancy’) are related to those in later parenthood, nor how parity influences this. As behaviours tend to track through adulthood,^28^ lower postpartum activity levels may remain into later adulthood, given the ongoing demands of parenthood. This may prove detrimental to the woman’s health, and impact familial physical activity (and health), given activity levels in mothers have been related to those of their (preschool-aged) children.^29,30^

Using data from the Southampton Women’s Survey, which provides unique data about collected from women before, during and after pregnancy, we aimed to determine how activity behaviours before/during pregnancy in women are related to device-measured activity levels in later parenthood. Additionally, we also explored how parity (i.e. whether a women had children or not before the index child’s birth) influenced associations through the inclusion of interaction terms.

## Methods

### Study design and setting

**Figure 1:**
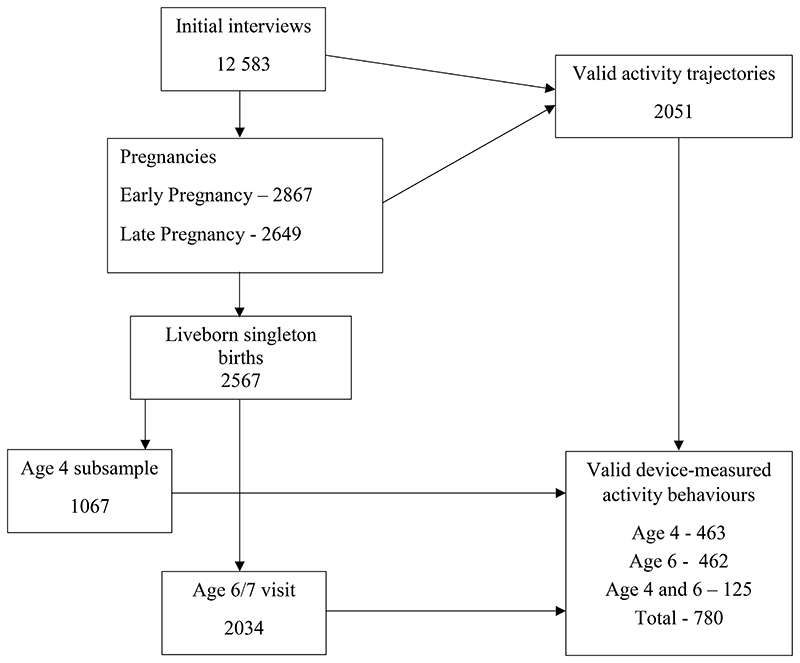
Participants in the SWS study

The Southampton Women’s Survey (SWS) is a population-based prospective cohort study based in Southampton, UK.^32^ The study assessed maternal diet and lifestyle before and during pregnancy, recruiting 12,583 non-pregnant women from General Practices in the Southampton area between 1998 and 2002. The survey provides novel data including (self-reported) measures of sitting, and moderate and strenuous physical activity before and at two time points during pregnancy. Subsequent singleton live births (n=3,158) were assessed at specific ages to observe how children’s pre-natal development interacts with their postnatal growth, and how both affect children’s risk factors for a range of future chronic diseases.^32^ Device-measured activity behaviours were also collected from women when her cohort child was 4 and 6 years old. Between March 2006 and June 2009 a sub-study was conducted to investigate physical activity in the index child at age 4 and their mothers. In total, 1,065 mother-child pairs were invited to participate,^33^ with the subsample of 4-year-old offspring recruited sequentially from later births in the SWS cohort. Children born after January 2000 were subsequently approached for an age 6-7 visit (March 2007 – August 2012), with mothers and children again both asked to wear an activity monitor (see Figure 1). Women also completed a questionnaire assessing demographic factors and physical activity correlates. Ethical approval for SWS data collection at all time points was granted by the Southampton and South West Hampshire Local Research Ethics Committee.

### Data collection

As part of the age 4 physical activity sub-study,^33^ and the 6-7 year visit (hereafter referred to as age 6), women were fitted with a combined heart rate and movement sensor (Actiheart, Cambridge Neurotechnology Ltd, UK) to measure their free-living physical activity. The monitor was secured to the chest and set to record at 60-second epochs to allow sufficient memory to record for 7 days. Participants were asked to wear the monitor continuously for seven days, including during sleep and water-based activities. Monitors were returned by secure post, along with the previously validated^34^ physical activity questionnaire.

### Outcome Measures

Only accelerometer data were used to define the accelerometer-based outcomes, with Actiheart data downloaded and processed using Stata 14/SE.^35^ At both time points, and for all participants, data periods of 100 minutes or more with zero-activity counts were removed, in line with common practice.^36^ Days with <600 minutes of recording were also removed, with 10 hours of activity being the minimum cut-off to define a valid day.^37^ All women with 2 or more days of valid physical activity data at age 4 and/or age 6-7 were included in analyses to maximise available data. Previous sensitivity analyses conducted in this cohort indicate that activity levels did not differ at age 4^29^ or age 6^30^ between those with ≥2 vs. ≥3 days of valid physical activity data. All recordings between 12am and 5am were removed, as were those between 10pm and 12am and 5-6am if they included more than 45 minutes of sedentary time, deemed to reflect hours spent sleeping. This method provides a conservative estimate of sleep time,^38^ whilst minimising an over-estimation of sedentary time in the evenings. Maximum daily wear time was therefore between 16-19 hours.

Two physical activity outcomes were then derived: time spent (minutes/day) sedentary (SED: <20 counts/minute) and in moderate-to-vigorous physical activity (MVPA: ≥400 counts/minute). Validated cutpoints were scaled with a conversion factor of 5, with Actiheart intensity thresholds equating to 100 counts for SED and 2000 for MVPA in the Actigraph 7164 accelerometer.^39,40^

### Exposure and confounding variables

At study recruitment (‘pre-conception’), during early (~12 weeks gestation) and late (~34 weeks) pregnancy, women were asked to complete a series of questions about their activity behaviours designed for this study. The validity and reliability of the physical activity questionnaire has not been formally examined, but empirical use in other cohorts shows that assessment of maternal physical activity relate to aspects of offspring body composition in plausible ways.^41^ For the sedentary analyses, we used the question “how many hours per day do you spend sitting?” to derive *hours per day women spent sitting* at each of the three time points before/during pregnancy. For the MVPA analyses, we used the reported duration (in hours per week) of a) moderate and strenuous exercise combined to derive *hours per week women spent in moderate-strenuous exercise*, and b) strenuous exercise only to derive *hours per week women spent in strenuous exercise*.

Using these data, we derived three trajectories for each woman reflecting: i) sitting, ii) moderate-strenuous exercise and ii) strenuous exercise before/during pregnancy. We took a median split of hours/day sitting, and hours/week for both exercise exposures, reported at pre-conception for woman’s baseline level of activity (sitting: ≤7/>7 hours/day; moderate-strenuous: ≤2.25/ >2.25 hours/week; strenuous: ≤0.25/ >0.25 hours/week). We then used this same value to classify women’s activity in early and late pregnancy, allocating them into a low or high category for each exposure. We combined these three dichotomous variables to generate a distinct trajectory for each exposure for each woman. So, for example, a woman reporting low sitting/exercise pre-conception, during early and late pregnancy was allocated a “0 0 0” trajectory for each of these exposures. Where applicable, several categories were combined to generate trajectory categories, and trajectory membership was composed of different women for the differing behaviours (e.g. a woman could be in the ‘consistent high’ sitting group but in the ‘decliner exercise’ group) (Table 1). Each of the three trajectories were used as exposure variables in analyses.

### Covariates

A range of confounding variables and competing exposures were also derived based on existing literature, using the Daggity software^42^ (see DAGs in Supplementary figures 1a/b). The accelerometer output provided hour, time of the day, weekday vs. weekend, and date, which were used to derive season of measurement [winter: December-February; spring: March-May; summer: June-August; autumn: September-November]. The level of educational qualifications a woman had obtained (GSCE or less/ A-level or HND/ Degree), her ethnicity (white/ non-white), her pre-conception BMI, parity (prior to conceiving the index child – 0/ 1 or more children), time taken to conceive the index child (in years), and whether she was living with a partner (yes/no) was derived from the pre-pregnancy questionnaire.

### Statistical analysis

Analyses were carried out using Stata/SE 14.^35^ Descriptive characteristics for women providing trajectories before/during pregnancy and physical activity data in later parenthood were derived, and differences in trajectory membership by demographic variables were explored for the analysis sample using appropriate tests. To assess sample representativeness, we compared women who provided physical activity data before/during pregnancy and in later parenthood (‘analysis sample’) with a) those who provided data before/during pregnancy only (‘trajectory sample’), and b) women who were recruited into the cohort but did not provide data before/during pregnancy.

Using women’s daily minutes spent sedentary or in MVPA as the outcome variables, two-level mixed effects (i.e. random intercept and random slope) models were used to model the association between each of the three trajectory variables and subsequent daily activity behaviour. Hierarchical models allow for variation across days (level 1) within women (level 2).^43^ Correlations between observations were accounted for by allowing the intercept to vary randomly between women (i.e. level 2). To take account of outcome data being included for women from age 4 and age 6 sweeps, a random slope for time (i.e. age 4 or age 6) was fitted, improving model fit based on likelihood ratio test (LRT). The random effect component therefore comprised intra-individual variation in activity across time and residual measurement error. We used an exchangeable correlation structure, suitable for repeated measures on the same person, as it specifies equal variances for random effects, and one common pairwise covariance. Due to non-normality, a log transformation of women’s MVPA was used for regression analyses. As a result, the regression coefficients were back transformed and are presented as the percentage change in MVPA per unit change compared to the baseline trajectory group.

Informed by DAGs mentioned earlier, models were minimally adjusted for appropriate confounders and competing exposures based on causal thinking (Supplementary figures 1a/b). In each model, an interaction term was included between trajectory and parity to determine whether having children prior to the index pregnancy differentially impacted the relationship between physical activity before/during pregnancy and in later parenthood.

We re-ran analyses for the moderate-strenuous exercise trajectory and MVPA outcome relationship, using a trajectory derived using guideline levels of moderate-strenuous exercise (i.e. ≤2.5/ >2.5 hours/week moderate-strenuous exercise at each time point rather than a median split). We also conducted sensitivity analyses, adjusting for wear time, to determine whether this influenced our findings. Finally, *posthoc* analyses were conducted to determine whether mode of delivery (i.e. natural vs c-section) or pregnancy complications differed across behaviour trajectories.

## Results

Valid trajectories were derived for 2051 women (‘trajectory sample’), of whom 780 had valid accelerometer data when their cohort child was age 4 and/or 6 (age 4 n=463; age 6 n=462; both n=125) and were included in analyses (‘analysis sample’). Women wore Actiheart monitors for a mean 13.9 (SD: 0.7) hours on 5.8 (SD: 1.4) days at age 4 and 14.3 (0.7) hours on 5.9 (SD: 1.5) days at age 6. Descriptive characteristics for the trajectory and analysis samples are presented in Table 2. Compared to the trajectory sample, those in the analysis sample were slightly older, but showed no differences in pre-conception BMI, educational attainment or ethnicity. The latter also reported slightly higher levels of sitting during pregnancy and lower levels of moderate-strenuous exercise (Supplementary Table 1). Women in the analysis sample were more likely to be white and have higher educational attainment than women initially recruited into the study.

Based on derived trajectories (Table 1), most women reported consistent (high or low) sitting before and during pregnancy (57%); 41% reporting consistent moderate-strenuous exercise; and the majority report decreasing (44%) or low (22%) levels of strenuous exercise. Average levels of reported sitting and exercise were consistent with the derived nomenclature of the trajectory groups (Supplementary Table 2). Women who already had children when pregnant with the index child were more likely to report consistently lower levels of sitting and less strenuous exercise before/during pregnancy than those for whom it was their first pregnancy. Women with higher educational attainment were more likely to report consistently high sitting and strenuous activity, but declining moderate/ strenuous exercise; women with lower educational attainment were more likely to report consistently low sitting and strenuous exercise, with increasing moderate/ strenuous exercise (Table 2). In later parenthood, accelerometry data indicated women were largely sedentary (for a mean (SD): 427.9 (151.0)minutes/day or 7.1 hours/day) and engaged in low levels of MVPA (median (IQR): 18.7 (10.2-29.0) minutes/day).

The results of the regression analyses are presented in Table 3. Compared to low sitters before/during pregnancy, women in all trajectory categories reporting high sitting in later pregnancy had higher daily sedentary time in later parenthood. For example, consistent high sitters before/during pregnancy were sedentary for 39.5 [23.26,55.82] minutes/day more in later parenthood than low sitters; those who increased their sedentary time during pregnancy (29.7 [13.03,46.29] mins/day) and those who were more sedentary both before and in later pregnancy were also more sedentary in later parenthood than consistent low sitters (43.7 [21.82,65.59] mins/day).

Compared to women with consistently low levels of moderate-strenuous exercise before/during pregnancy, those with consistently high moderate-strenuous exercise engaged in 22% more MVPA in later parenthood (GMR: 1.22 [95% CI: 1.02, 1.47]). Similarly, women who engaged in consistently high (vs consistently low) levels of strenuous activity before/during pregnancy were 25% more likely to engage in MVPA in later parenthood (GMR: 1.25 [1.04,1.51]). In addition, those starting from a higher baseline of strenuous activity preconception (‘decreasers’; 1.22 [1.06,1.41]; variable (low early): 1.41 [1.11,1.80]) also engaged in more MVPA in later parenthood.

LRTs favoured simpler models for all exposure-outcome associations, indicating that there was no interaction between parity and each of the three activity trajectories. Interaction terms were therefore not included in final analyses (i.e. p<0.05, our *a priori* significance level). Sensitivity analyses adjusting for wear time indicated findings were comparable in magnitude and statistical significance (data not shown). *Posthoc* analyses indicated there were no differences in trajectory membership in later parenthood by mode of delivery or pregnancy complications (Supplementary Table 1).

## Discussion

This is one of the first studies to explore how activity behaviours reported before/during pregnancy are associated with those in later parenthood. Most women reported stable levels of sitting, with consistently high sitting before/during pregnancy (compared to low) associated with approximately 40 minutes/day more device-measured sedentary time in later parenthood. In general, women with higher sitting in later pregnancy had higher levels of sedentary time in later parenthood. Women’s trajectories of moderate-strenuous exercise were more variable, but the majority had decreasing levels of strenuous activity during preconception and pregnancy. Those with consistently higher moderate-/strenuous exercise (vs low) engaged in ~22% more MVPA as parents. In addition, trajectories with higher levels of strenuous activity pre-conception were associated with more MVPA. Given that in general, women tend to have lower physical activity levels after becoming a parent,^26,27^ intervention strategies targeted at preconception to encourage higher intensity activity, and before/during pregnancy to reduce sitting, may allow women to enter parenthood with more favourable activity behaviours, and therefore be more likely to rebound to positive habits in later parenthood.

Almost a third of women in this sample (~30%) reported >7 hours per day sitting across the pre-conception and pregnancy period, with a further 20% reporting increasing their sitting time to >7 hours during pregnancy. This was in combination with generally low reported levels of strenuous activity (median: 15 min/week). This is perhaps unsurprising given the physical demands that pregnancy places on the body,^44^ but is important given the independent associations noted between health, sedentary time and higher intensity activity.^45,46^ In all trajectory groups with high levels of sitting in later pregnancy, regardless of starting point,women had far higher levels of sedentary time in later parenthood (~30-40 minutes/day). Levels of sitting during pregnancy were higher here than have been reported in other cohorts,^21^ and in the short term may result in poorer metabolic indicators (e.g. LDL Cholesterol) for pregnant mothers, and larger abdominal size and higher birth weight in babies.^7^ Longer term, differences in device-measured sedentary time are potentially clinically significant, as sedentary time, regardless of meeting physical activity guidelines, is independently associated with poorer health outcomes,^46^ and women (with small children) are at greater risk of cardiovascular disease.^47,48^ Although *post hoc* analyses indicated that there were no differences in trajectory membership by mode of birth or pregnancy complications, it is possible that pregnancy discomfort and subsequent ongoing health issues may in part explain these finding. Nevertheless, this work hints that sitting time may be high in British women during before/during pregnancy, and encouraging women to reduce their sitting time, even in favour of higher intensity ‘light’ activity, particularly in later pregnancy, may bring both short- and longer-term benefits for both mothers and children.

Consistently higher levels (vs low) of moderate-/strenuous exercise before/during pregnancy were associated with engaging in over 20% more MVPA in later parenthood, and this pattern was also true for all groups in the higher category of strenuous exercise pre-conception. This suggests that even small amounts of higher intensity exercise, particularly before pregnancy, may be indicative of positive activity habits which women return to in later parenthood, likely conferring benefits into later parenthood and beyond.^45^ Continuing to be active during pregnancy, at higher intensities where safe to do so, is also related to better birth outcomes,^3,6^ and may allow women to maintain their fitness and activity habits, whilst managing pregnancy weight-gain. In addition, although evidence is sparse, higher physical activity levels in women during the pre-conception period have been suggested to have benefits for fertility and fecundability.^49,50^ This therefore argues for greater focus on developing interventions to encourage women to engage in physical activity throughout pregnancy, and at higher intensity levels pre-conception, to garner wide-ranging benefits into later life.

Interestingly, associations between activity behaviours before/during pregnancy and subsequent device-measured were the same for woman who did and did not already have children when pregnant. Overall, consistency appeared to be the strongest predictor of later behaviour, with stable levels of higher sitting and exercise associated with the equivalent device measured behaviour in later parenthood. Activity behaviours in the general population track through adulthood,^28^ and so it may be that pregnancy and the postpartum period signal a relatively brief but potentially important perturbation in behaviours during adulthood. Differences in sitting and strenuous exercise trajectories by parity were however apparent, with women who already had children being more likely to report consistently lower levels of sitting and strenuous activity before/during pregnancy. This aligns with previous work showing that women who have children are less sedentary and engage in more light physical activity.^51^ The latter was also identified in this sample of women when their index child was aged 4 years.^33^ Women’s activity levels may differ as the index child ages, and indeed by the ages and number of children in the household,^31^ but ultimately, the impact of children is the same on later behaviours, regardless of number. Given the strong behavioural nature of sitting and indeed higher intensity activity, provision for all women to positively change their activity behaviours as they prepare for and enter pregnancy is therefore required, though the types of support women may seek to facilitate this will likely differ based on her family circumstances.

Finally, this work highlights the importance of collecting data about, and including metrics of, activity behaviours in preconception and pregnancy pathways. Despite guidelines recommending physical activity and limited sitting for women of childbearing age during pregnancy and postpartum,^2^ limited population-level evidence suggests that women fail to meet guidelines before, during and after pregnancy.^13,14,16^ Without high-quality nationally representative surveillance metrics, we face an ongoing data gap pertinent to both maternal and child health. At present, it is impossible to understand the scale of the inactivity problem, where interventions should be targeted and who may benefit most. However, given the detrimental impact to women’s and infants’ health of high levels of sitting and low physical activity during pregnancy and beyond,^7,46–48^ it is vital we develop a better understanding of activity behaviours in this population subgroup to support positive behaviour change.

### Strengths and Limitations

This study is one of the first to describe, in a large population-based sample of British women, the association between activity behaviours reported prior to and during pregnancy, and subsequent device-measured activity behaviours in later parenthood. Using time-stamped accelerometer data, we accounted for variation in women’s daily device-measured physical activity during the measurement period to build a detailed picture of activity behaviours in women in later parenthood. The use of longitudinal data and DAGs are further strengths of the study, to help identify causal associations. Sitting, moderate and strenuous activity levels before/during pregnancy were self-reported by women, which may have resulted in biased estimates. It is plausible however that the bias in reporting would be consistent within women, and as behaviours were reported prospectively, they are not subject to recall bias. Although binary classification of high/low sitting and exercise was based on pre-defined guidance for recommended levels, use of binary categories to generate the trajectories is a reductive technique. Care was taken to ensure that mis-classification using this method was minimised, but inevitably as a result of the classification process, some information will be lost. Sitting is not a direct equivalent for sedentary time, nor exercise for MVPA, but using the former as proxies for the latter allowed us to build a picture of lesser studied activity during a key time in women’s and children’s lives. Although we adjusted for DAG-derived confounders, residual confounding cannot be ruled out. Device-measured physical activity data were collected in the women up to 2012, but are likely still relevant considering that physical activity levels amongst adults in high-income western countries have continued to decline.^52^

The analysis sample were slightly older and reported more sitting and less exercise than the trajectory sample, which suggests the former were not necessarily more likely to be included in the device-based measures because they were more active than their peers. The analysis sample contained women who were likely to be white and have higher educational attainment than those originally recruited. Higher socio-economic status is associated with increased leisure time, but decreased occupational activity in adults.^53^ However, as women’s employment data were not collected at the age 4 and 6-7 year visits, we can’t comment on how this might have impacted our findings. Nevertheless, as with any cohort, attrition may have resulted in those in the analysis sample being somewhat different from the original cohort and this should be borne in mind.

## Conclusions

In a population-based sample of British women, we found that trajectories of sitting and exercise before/during pregnancy are associated with device-assessed sedentary time and MVPA respectively in later parenthood, suggesting that activity behaviours pre/pregnancy predict those in later parenthood. Given the impact of these behaviours on longer-term maternal and child health, interventions are required to reduce sitting before/during pregnancy, and to encourage higher intensity activity pre-conception, to benefit both mothers and their children during pregnancy and beyond.

## Supplementary Material

Supplementary Figure 1a

Supplementary Figure 1b

Supplementary File

Supplementary Tables

## Figures and Tables

**Table 1:** Derivation of exposure trajectories and proportion of women in each group by Trajectory (n=2051) and Analysis (n=780) samples

*Hours/day sitting or Hours/week in moderate/strenuous exercise*	Trajectory			Sitting *Hours/ day*		Moderate/Strenuous exercise *Hours/week*		Strenuous exercise only *Hours/week*	
	Median split (0= low; 1= high)			Trajectory^#^ n= 2051	Analysis^#^ n=780	Trajectory^#^ n= 2051	Analysis n=780	Trajectory^#^ n= 2051	Analysis^#^ n=780
	Pre-Concept	Early Preg.	Late Preg.						
Consistent - low	0	0	0	540 (26.3)	183 (23.5)	596 (29.1)	242 (31.0)	461 (22.7)	174 (22.4)
Consistent - high	1	1	1	625 (30.5)	244 (31.3)	244 (11.9)	88 (11.3)	265 (13.0)	112 (14.4)
Increasers (early pregnancy)	0	1	1	402 (19.6)	161 (20.6)	205 (10.0)	70 (9.0)	106 (5.2)	50 (6.4)
Increasers (late pregnancy)	0	0	1						
Decreasers (early pregnancy)	1	0	0	233 (11.4)	83 (10.6)	706 (34.4)	277 (35.5)	932 (45.8)	340 (43.8)
Decreasers (late pregnancy)	1	1	0						
Variable (low early pregnancy)	1	0	1	147 (7.2)	66 (8.5)	150 (7.3)	50 (6.4)	123 (6.1)	48 (6.2)
Variable (high early pregnancy)	0	1	0	104 (5.1)	43 (5.5)	150 (7.3)	54 (6.9)	145 (7.1)	53 (6.8)

# Indicates difference in group membership by parity.

**Table 2:** Descriptive characteristics of women in Trajectory and Analysis samples

	Trajectory (n=2051)	Analysis (n=780)
		
Pre-conception BMI (Mean (SD))	25.4 (4.7)	25.1 (4.4)
Ethnicity (non-white, n(%))	67 (3.3)	24 (3.8)
Educational attainment (n(%))^^*±^		
GSCE or less	814 (39.8)	298 (38.2)
A-level / HND	770 (37.6)	304 (39.0)
Degree	462 (22.6)	178 (22.8)
Living with partner (n(%))	1641 (80.1)	646 (82.8)
Age at study entry (Mean in years (SD))	28.0 (3.8)	28.4 (3.7)
Parity (n(%))^^±^		
Nulliparous	1027 (50.1)	407 (52.2)
Multiparous	1023 (49.9)	373 (47.8)
		
**Outcome data (mins/day)**		
SED: mean (SD)	-	427.9 (151.0)
MVPA: mean (SD)	-	22.2 (16.8)
MVPA: median (IQR)	-	18.7 (10.2-29.0)

^ Differs significantly across sitting trajectory;

* Differs significantly across moderate/ strenuous exercise trajectory;

± Differs significantly across strenuous exercise trajectory;

GCSE: General Certificate of Secondary Education; HND: Higher National Diploma; SED: Sedentary; MVPA: Moderate-vigorous physical activity; SD: standard deviation; IQR: Interquartile range.

**Table 3:** Associations between behaviour trajectories before/ during pregnancy and subsequent device-measured activity behaviours

Parenthood outcome	Sedentary	MVPA		MVPA
	β minutes /day [95%CI]	GMR^^^ [95%CI]		GMRA^^^ [95%CI]
Pre/pregnancy exposure	Sitting	Moderate/strenuous exercise median split	Moderate/strenuous exercise guidelines	Strenuous exercise only
				
Trajectory category **(ref: Consistent low)**				
Consistent high	**39.5 [23.26,55.82]**	**1.22 [1.02, 1.47]**	**1.26 [1.03,1.55]**	**1.25 [1.04,1.51]**
				
Decliners	12.5 [-7.67,32.59]	1.03 [0.90,1.17]	1.02 [0.90,1.16]	**1.22 [1.06,1.41]**
				
Increasers	**29.7 [13.03,46.29]**	1.13 [0.92,1.39]	1.11 [0.90,1.38]	1.05 [0.83,1.33]
				
Variable Low preconception	7.2 [-18.82,33.16]	1.03 [0.82,1.29]	1.18 [0.94,1.47]	1.04 [0.82,1.32]
				
Variable High preconception	**43.7 [21.82,65.59]**	1.20 [0.96,1.51]	1.12 [0.90,1.41]	**1.41 [1.11,1.80]**

^ Exponentiated b or Geometric Mean Ratio (GMR):

any deviation from 1 indicates a % change in category MVPA per unit change relative to MVPA in the reference category. Analyses adjusted for pre-conception BMI, maternal qualifications, age, parity and living with a partner at index child’s birth, season and time of the week. Bold indicates confidence interval does not overlap 1.
