# Supplementary figures and images for: Activity behaviours before and during pregnancy are associated with women’s device-measured physical activity and sedentary time in later parenthood: a longitudinal cohort analysis
Source: J Phys Act Health. Author manuscript; Available in PMC 2023 Oct 7. (PMC7615174; doi:10.1123/jpah.2022-0630)

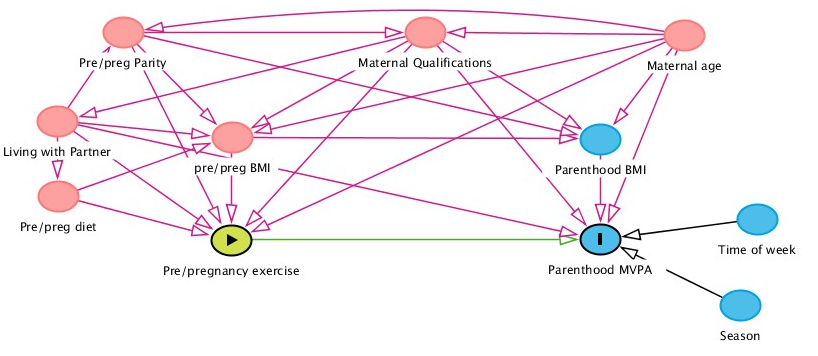

Supplement: Supplementary Figure 1a [file EMS188202-supplement-Supplementary_Figure_1a.png]

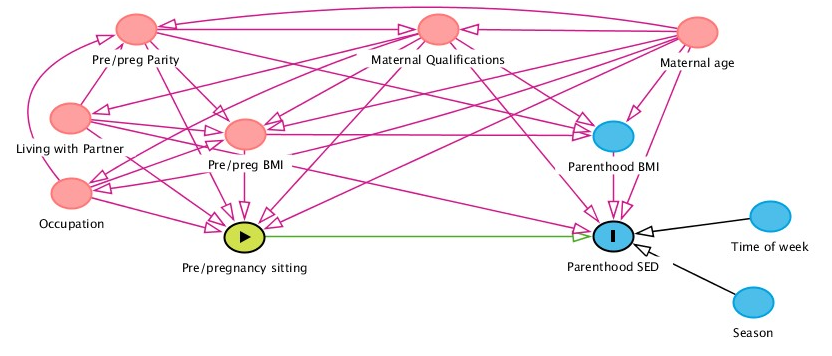

Supplement: Supplementary Figure 1b [file EMS188202-supplement-Supplementary_Figure_1b.png]
