# Supplementary material for: Activity behaviours before and during pregnancy are associated with women’s device-measured physical activity and sedentary time in later parenthood: a longitudinal cohort analysis
Source: J Phys Act Health. Author manuscript; Available in PMC 2023 Oct 7. (PMC7615174; doi:10.1123/jpah.2022-0630)

Supplementary Figure 1a: DAG for associations between pre/pregnancy exercise and device-measured physical activity in later parenthood.

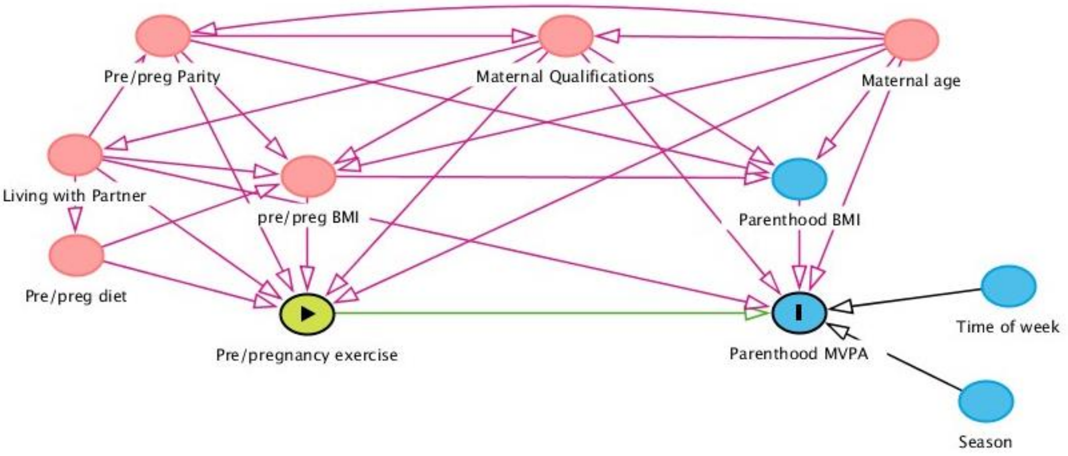

Supplementary Figure 1b: DAG for associations between pre/pregnancy sitting and device-measured sedentary time in later parenthood.

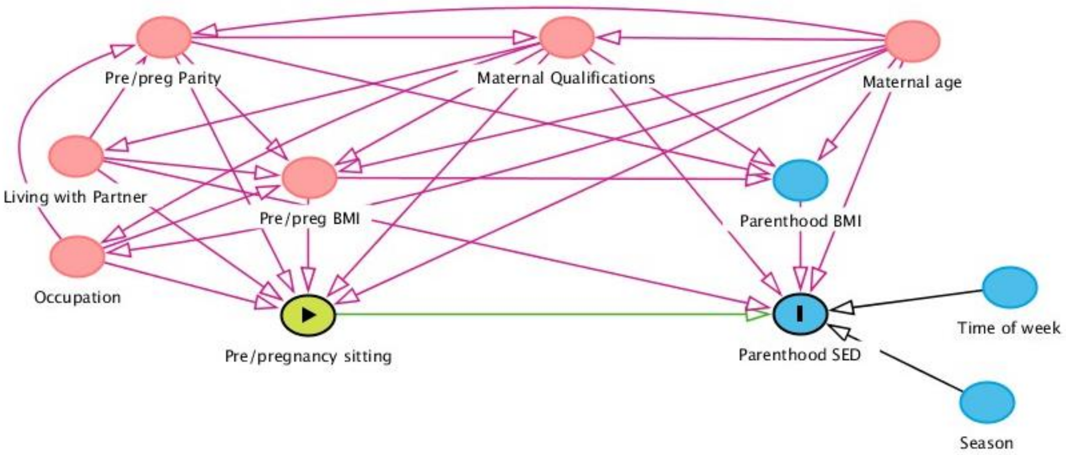

Analyses (minimally) adjusted for maternal pre-conception BMI, qualifications, living with partner, age and parity at child's birth, season and time of the week.

DAG KEY

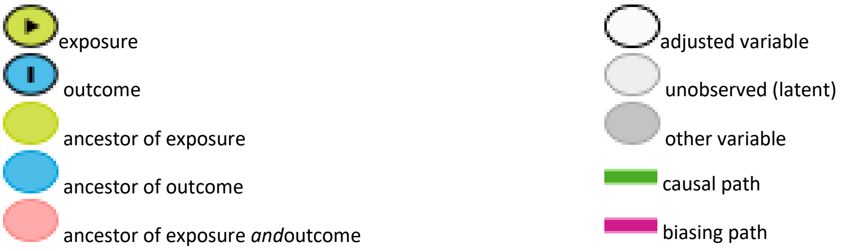

Supplement: Supplementary File [file EMS188202-supplement-Supplementary_File.pdf]
