# Supplementary material for: Activity behaviours before and during pregnancy are associated with women’s device-measured physical activity and sedentary time in later parenthood: a longitudinal cohort analysis
Source: J Phys Act Health. Author manuscript; Available in PMC 2023 Oct 7. (PMC7615174; doi:10.1123/jpah.2022-0630)
Supplement: Supplementary Tables [file EMS188202-supplement-Supplementary_Tables.pdf]

Supplementary Table 1: Average times reported time sitting and in moderate/strenuous exercise before/during pregnancy

	Trajectory sample (n=2051)			Analysis sample (n=780)		
Reported time	Pre-conception	Early Pregnancy	Late Pregnancy	Pre-conception	Early Pregnancy	Late Pregnancy
Sitting^ (hr/day)	7.4 (3.1)	7.4 (3.0)	7.9 (2.9)	7.6 (3.1)	7.7 (2.9)*	8.1 (2.8)*
Moderate/ strenuous exercise (hrs/week)	3.6 (3.7)	2.5 (3.4)	2.0 (3.0)	3.5 (3.6)	2.4 (3.4)	1.9 (2.9)
Strenuous^ exercise (hrs/week)	1.1 (1.7)	0.46 (1.2)	0.22 (0.8)	0.97 (1.6)	0.53 (1.4)	0.21 (0.7)

* Indicates difference between trajectory and analysis sample (*t-test* $p<0.05$)
^ Indicates difference in mean levels by parity (*t-test* $p<0.05$)
Posthoc tests indicated no significant difference in time spent sitting or in moderate-/strenuous exercise by mode of delivery or pregnancy complications.

Supplementary Table 2: Average times reported time sitting and in moderate/strenuous exercise by trajectory category

	Consistent low	Consistent high	Decliners	Increasers	Variable (Low preconception)	Variable (High preconception)
	All values are median [IQR]					
**Sitting (hours/day)**						
Preconception	5.0 [3.9, 6.0]	10.0 [9.0, 12.0]	9.0 [8.0, 10.0]	5.5 [4.0, 6.5]	5.5 [4.0, 6.0]	9.0 [8.0, 10.0]
Early pregnancy	5.0 [4.0, 6.0]	10.0 [9.0, 12.0]	7.0 [5.5, 8.5]	7.0 [5.0, 9.0]	8.0 [8.0, 10.0]	6.0 [5.0, 7.0]
Late pregnancy	5.0 [4.0, 6.0]	10.0 [9.0, 12.0]	6.0 [5.0, 7.0]	9.0 [8.0, 10.0]	6.0 [5.0, 7.0]	9.0 [8.0, 10.0]
**Moderate/ strenuous exercise (hours/week)**						
Preconception	0.79 [0.21, 1.5]	6.2 [3.8, 9.3]	4.8 [3.4, 7.5]	1.0 [0.25, 1.7]	1.1 [0.35, 1.6]	5.4 [3.7, 8.5]
Early pregnancy	0.38 [0.0, 1.0]	7.0 [3.8, 10.0]	1.5 [0.38, 3.4]	1.5 [0.25, 4.0]	4.5 [3.0, 6.9]	1.2 [0.30, 1.5]
Late pregnancy	0.25 [0.0, 0.80]	5.3 [3.5, 9.0]	0.75 [0.06, 1.5]	4.7 [3.5, 7.0]	0.75 [0.10, 1.5]	4.7 [3.1, 7.0]
**Strenuous exercise (hours/week)**						
Preconception	0.0 [0.0, 0.0]	1.1 [0.50, 2.6]	1.00 [0.25, 1.5]	0.0 [0.0, 0.0]	0.0 [0.0, 0.0]	0.50 [0.25, 1.5]
Early pregnancy	0.0 [0.0, 0.0]	0.75 [0.25, 1.5]	0.0 [0.0, 0.0]	0.0 [0.0, 0.0]	0.50 [0.12, 1.5]	0.0 [0.0, 0.0]
Late pregnancy	0.0 [0.0, 0.0]	0.50 [0.12, 1.5]	0.0 [0.0, 0.0]	0.25 [0.10, 0.75]	0.0 [0.0, 0.0]	0.25 [0.10, 1.3]
